# Physiology of Oral Hygiene*Read before the Manchester Odontological Society, March, 1920. Reprinted from *The Dental Record*, November, 1920.

**Published:** 1920-12

**Authors:** J. Sim Wallace

**Affiliations:** Formerly Lecturer on Dental Surgery and Pathology at the London Hospital


### PHYSIOLOGY OF ORAL HYGIENE.*

BY J. SIM WALLACE, D.SC., M.D., L.D.S.,
Formerly Lecturer on Dental Surgery and Pathology at the London Hospital.

To understand properly the physiology of oral hygiene it is necessary to refer to the physiology of mastication, for although the chief function of mastication is the preliminary preparation of food in order to facilitate its digestion, it has a secondary function in that when suitable foods are masticated it cleans the teeth and facilitates the action of the saliva in doing likewise. It should be noted, however, that many foodstuffs which are consumed at the present day are hardly subjected to the process of mastication at all. The food is simply taken into the mouth, receives a general squash between the teeth or between the dorsum of the tongue and the hard palate, and is then swallowed. This method of mastication, if mastication it can be called, is, as a rule, adopted for custards, fine meal porridge, soft puddings, and soft, nonfibrous foods generally.

When there is a certain amount of crisp, spongy or fibrous matter in the foodstuff, then the process is essentially different, and mastication is performed in a more thorough manner. In this latter case the food is crushed and torn between and heaped on to the masticating surfaces of the teeth by the muscular contractions of the tongue, cheeks and lips, and by the motions of the lower jaw. During comminution between the teeth, the juices of the foodstuffs, the saliva which becomes incorporated, and the suspended nonfibrous part, are pressed out from the fibres and gradually collect during the process on the middle of the dorsum of the tongue, which is grad-

* Read before the Manchester Odontological Society, March, 1920. Reprinted from *The Dental Record*, November, 1920.

ually hollowed out for the reception of such food, and this part is then swallowed. The fibrous part of the food, however, is subjected again and again to the crushing and disintegration between the teeth.

When for any reason mastication is not performed on one side of the mouth, the teeth on that side become coated with mucus, tartar, and food debris. It is evident, therefore, that mastication of fibrous food is conducive to dental hygiene.

Now let me direct your attention more particularly to mucus and saliva in regard to oral hygiene. I shall do so at some length because the subject is never even mentioned in physiological text books. Physiologists hardly seem even yet to realize that oral hygiene is a physiological process, and that all the glands opening into the mouth, together with their secretions, are specially adapted for the maintenance of oral hygiene, and that these secretions are scarcely, if at all, of importance for any other function. From the point of view of the oral hygienist the supposed function of the mucus for lubricating the bolus of food so as to facilitate its passage down the œsophagus is whimsical, for inasmuch as the function of mastication is to comminute the food, and reduce anything in the nature of a bolus to a liquid or semi-liquid state before its passes to the back of the dorsum of the tongue, there is no bolus to lubricate. The mucus and saliva are thoroughly incorporated with the food, and consequently the semi-liquid food does not require lubrication. Just as the function of the mucus in the bronchial tubes is to keep these tubes clean, so the essential function of the oral mucus is to keep the mouth clean. Mucus is a liquid, tenacious and ropy in its nature, which under certain circumstances clings to hard substances. Chemically, perhaps, its best-known characteristic is that it forms a flocculent precipitate when treated with acids. These properties of mucus are well known. The following quotation from the work of Professor W. E. Gies and his collaborator, Dr. Lothrop—great authorities on mucus—will indicate some further characteristics: "Mucin occurs in saliva, and apparently also on dental surfaces, primarily as acid salts in concentrated colloidal solution. . When viscid mucinous coatings are treated with basic material, such as carbonate of an alkali or an earthy element, the mucin mass becomes superficially more smeary and slippery by reason of the production of more soluble mucin salts at the surface. Complete mechanical removal of a mucin plaque from a tooth is facilitated by the addition of a basic material that renders the mucin superficially more viscous, but the slippery surface thus produced may make the application of considerable friction necessary for the detachment of the plaque. On the other hand, when a viscid mucinous deposit is treated with acid material, the mucin mass is completely disintegrated by a curdling or aglutinative process, the particles are devoid of adhesiveness to smooth surfaces, stickiness disappears because of the precipitation of caseous mucin itself, and the entire disorganized mass may be readily flushed away."

A consideration of these properties of the mucus leads us to the conclusion that it is quite ingeniously suited for keeping the mouth free from the undue lodgment of food particles, when the foods are not converted by artificial means into some bland pap-like form which stultifies efficient mastication, negatives "considerable friction," and precludes the possibility of having it disintegrated so that it may be readily flushed away. It matters but little whether the chemical reaction of the food is neutral or alkaline, for during the mastication of food the saliva always becomes alkaline if in its resting state it is acid, and the slippery state of the mucous coating on the teeth (and gums) is assured, while the ropiness of the muces is left unimpaired.

On the other hand, if the food is more acid than can be neutralized by the copious flow of alkaline saliva, which it stimulates, the mucus plaques themselves are disintegrated and easily removed by the saliva and mucus after the acid has been swallowed. Thus, then, the mucus has not only the power of facilitating the removal of food particles and shreds which have been disintegrated by mastication, but it has also, under certain dietetic conditions, the property of being disintegrated and carrying its own disintegrated self away. Under unsatisfactory dietetic conditions—that is, when the food is habitually alkaline and so soft as not to stimulate mastication—the mucous coating on the teeth may remain too long stagnate, and become infiltrated with the salts of the saliva—that is to say, tartar may be formed; or, if the food is of a carbohydrate nature and of a sticky character, it may be rapidly converted into lactic acid by the bacteria of the mouth, decalcification of the enamel may take place, and dental caries may be initiated. Should, however, the mucus not be saturated with carbohydrates of an impermeable and easily fermentable nature, the bacteria of the mouth slowly disintegrates the mucous coatings on the teeth and facilitate its removal on those surfaces of the teeth where it has not been removed by disintegration or considerable friction. It is quite possible too that the ptyalin in the saliva may digest the carbohydrate radical in the mucus, just as trypsin does. Why otherwise should we have a saliva rich in ptyalin poured out when sugar is taken in the mouth?

When we consider these facts we see that mucus is ingeniously devised for oral hygiene, and it is not devised for the lubrication of the bolus of food to facilitate its transmission to the stomach, nor is it devised, as has been suggested, to keep the numerous boluses of food in the stomach from the action of the hydrochloric acid, so that the ptyalin may have a chance of converting the starch in the boluses. Indeed, it seems much more probable that the thorough incorporation of food with mucus, facilitates the penetration of the hydrochloric acid throughout the whole of the contents of the stomach after a meal, and possibly this is one of the reasons why a meal of such a physical consistency as demands mastication is more quickly and satisfactorily digested than a meal of a similar nature which has been reduced to a pap-like form before it is taken into the mouth.

### SALIVA

It seems to me more than probable that physiologists will, in the near future, require to reconsider their teachings with regard to the functions of the saliva. Hitherto they have looked upon it as the first of the digestive juices, and the salivary glands have been regarded as the first of the digestive glands.

In recent years, however, we have studied physiological problems because of their importance with regard to human welfare, and we have come to the conclusion that to all intents and purposes the saliva is not a digestive juice, but that it is practically wholly for the purposes of oral hygiene. The older physiologists have based their ideas on the fact that ptyalin has the power of converting cooked starch into achro-dextrine and maltose. But many questions arise which would appear to throw considerable doubt upon regarding this as the chief function of saliva. That the digestion of uncooked starch is practically negligible is admitted by them; indeed, we are told that ptyalin does not digest uncooked starch (Halliburton, F. A. Bainbridge and J. Ackworth Menzies). If this be so we may ask why ptyalin exists in the saliva of animals at all. Is it to be conceded that the saliva is not a digestive juice in animals? Again, it has never been contended that ptyalin digests sugar, but sugar stimulates a relatively violent flow of saliva rich in ptyalin. Why, too, should ptyalin exist in the saliva from birth, as we are told by some physiologists?

Oral hygiene is certainly necessary with a milk diet, but are we to believe that ptyalin is there to digest starch which normally is not present?

Again, why is starch, immediately it has been masticated and mixed with the saliva, transferred at once to the stomach, where the conditions are so frequently such that the digestion of starch by the ptyalin is immediately arrested, or if it is not immediately arrested, it soon becomes so? Physiologists have, of course, long recognized this supposed delinquency of Nature, and attempts have been made to explain how ptyalin may have some reasonable time to digest starch before the contents of the stomach become sufficiently acid to arrest the action of the ptyalin. Thus for example, Messrs. Bainbridge and Menzies say: "The food, after a meal is taken, forms a compact mass in the stomach, and the hydrochloric acid of the gastric juice penetrates comparatively slowly into this mass." They evidently think not only that the food passes down the œsophagus in bolus form, but that the various boluses become one glorified bolus in the stomach! It is significant, too, that these physiologists never seem to refer to the fact that the contents of the stomach may be acid from the very beginning of a meal. A meal may commence with acid hors d'œuvre, the bit of fish which follows may be served with lemon juice, meat may be eaten with salad well seasoned with vinegar, and stewed or raw fruit or both may complete the meal with a still further supply of acid, while acid mineral or alcoholic beverages, which are sometimes supposed to stimulate digestion, may, and very frequently do, accompany the meal at various stages. Nevertheless, I do not know that we are told that acids taken in these ways interfere with the digestion of starch except among dyspeptics. Indeed, Professor Pawlow, speaking of the reaction of the food, says: "It is apparent that acidity enjoys a special preference in the human taste, . . . These facts are all physiologically comprehensible when we know that an acid reaction is not only necessary for an efficient action of the peptic ferment, but is at the same time the strongest excitant of the pancreatic gland.''

Another point on which there seems to be confusion is with regard to the medium most suitable for the digestion of starch. According to Messrs. Bainbridge and Menzies, ''the digestive action of ptyalin on starch is most energetic in a neutral medium.'' According to Foster, ''the action of saliva on starch is favored by a slightly alkaline medium.'' It is curious how this is always referred to as if the slight difference in the reaction of the saliva were really of considerable importance in digestion. I have frequently tested the reaction of saliva between meals, but certainly am unable to draw the inference that the digestion of starch is at all influenced in any practical way by the reaction of saliva. On the other hand the fact that the saliva always becomes very distinctly alkaline when food is eaten, and for some considerable time after it is swallowed, seems to the older physiologists unworthy of being mentioned. To the amateur physiologist, who thinks that oral hygiene should be at least as common in man as it is in animals, all these apparently confusing and inexplicable facts have a simple explanation. The marked alkalinity of the saliva when food is taken has a significance in that it is important that it should have that reaction while the mucus is specially required for removing food particles from the mouth. If the saliva were sufficiently acid the mucus could not function in this way. Moreover, the acids which so commonly accompany foods might decalcify the teeth. On the other hand, the reaction of the saliva between meals is of practically no importance at all, because the conversion of starch in the mouth into soluble starch proceeds to all intents and purposes sufficiently satisfactorily whether the saliva be neutral, or faintly acid, or faintly alkaline to litmus. It has been the subject of much contention, possibly on account of its lack of importance, whether acidity or alkalinity of the saliva was the more unfavorable to the inception of dental caries. Here let me say, although some physiologists affirm that ptyalin does not act on uncooked starch, others (for example, Michael Foster) say that raw unboiled starch undergoes the same change as boiled starch, "though more slowly."

Near the beginning of this paper I suggested a use for the ptyalin when sugar is taken into the mouth, and to those who appreciate why sugar is liable to cause dental caries, the reason for a copious flow of saliva when sugar is eaten is obvious. Similarly the desirability of a copious flow of alkaline saliva when acids are taken into the mouth is equally obvious if we value the preservation of the teeth; but why an alkaline saliva should be thought desirable for digestion when acid is "necessary for the efficient action of the peptic ferment, and at the same time the strongest excitant of the pancreatic gland," we fail to see.

While we are regaled with the importance of ptyalin for the digestion of starch, little is told us of the functions of the salts in the saliva, but they, too, are obviously of importance from the point of view of oral hygiene and the preservation of the teeth. Dr. Joseph Head made the important discovery that when the superficial layer of enamel of the teeth has been slightly softened by acid, this enamel can be rehardened by immersion in saliva, and thus the salts in the saliva have an important restorative value as well as being prophylactic in function.

The saliva contains something more—amœboid phagocytic cells—the so-called salivary corpuscles. They may be regarded as scavengers of the mouth, and, as far as we can surmise, their function is simply oral hygiene. We are told that they are probably derived from the tonsils (Halliburton) and although we might expect that lymphoid cells derived from the tonsils would pass down the throat rather than come forward and mix with the saliva, I at least have not seen reference to any experiment or observations upon which this idea is founded.

The late Professor G. V. Black made a careful examination of the lymphatics surrounding the necks of the teeth, and refers to a portion of the connective tissue in immediate conjunction with the tooth, which is not covered by the epithelium, and says, "It seems to be through this space that the cells—the so-called salivary corpuscles found under the free border of the gingiva—pass.

"These may be found at any time under the healthy gingivus, and their numbers are augmented with every irritation of the membrane." It has always appeared to me that the only satisfactory method of controlling the bacteria in the sulcus between the gingival margin and the tooth, is by means of these phagocytic corpuscles. If we admit that the salivary corpuscles exude into this sulcus, we observe at once their importance in oral hygiene, and, in passing, it may be said, the value of masticating food of such a nature as will cause an irritation, or at least stimulation, of the periodontal membrane, and thus assure a sufficient supply of these scavengers and thereby prevent the onset of pyorrhea.

All things considered, it appears that the function of the saliva is, par excellence, oral hygiene, and no matter whether the food passes into the mouth and down the œsophagus in the ordinary way or whether the food passes up from the stomach and out of the mouth, it may in general be said that the saliva is secreted in quality and quantity proportionate to the needs of the food, to expel the food as completely as possible from the mouth. I need hardly mention that although a copious flow of saliva is induced by vomiting, even physiologists of the old school could scarcely claim that under these circumstances the saliva was to be regarded as the first of the digestive juices.

I have put forward the preceding observations to induce physiologists to take up this subject immediately, and to give an authoritative lead. For without this the rank and file of the medical profession cannot be expected to teach the public the new ideas consistenly.

